# The effect of dietary fat consumption on Alzheimer’s disease pathogenesis in mouse models

**DOI:** 10.1038/s41398-022-02067-w

**Published:** 2022-07-22

**Authors:** Anna Amelianchik, Lauren Sweetland-Martin, Erin H. Norris

**Affiliations:** Patricia and John Rosenwald Laboratory of Neurobiology & Genetics, The Rockefeller University, 1230 York Avenue, New York, NY USA

**Keywords:** Pathogenesis, Diseases, Neuroscience

## Abstract

Alzheimer’s disease (AD) is a fatal cognitive disorder with proteinaceous brain deposits, neuroinflammation, cerebrovascular dysfunction, and extensive neuronal loss over time. AD is a multifactorial disease, and lifestyle factors, including diet, are likely associated with the development of AD pathology. Since obesity and diabetes are recognized as risk factors for AD, it might be predicted that a high-fat diet (HFD) would worsen AD pathology. However, modeling HFD-induced obesity in AD animal models has yielded inconclusive results. Some studies report a deleterious effect of HFD on Aβ accumulation, neuroinflammation, and cognitive function, while others report that HFD worsens memory without affecting AD brain pathology. Moreover, several studies report no major effect of HFD on AD-related phenotypes in mice, while other studies show that HFD might, in fact, be protective. The lack of a clear association between dietary fat consumption and AD-related pathology and cognitive function in AD mouse models might be explained by experimental variations, including AD mouse model, sex and age of the animals, composition of the HFD, and timeline of HFD consumption. In this review, we summarize recent studies that aimed at elucidating the effect of HFD-induced obesity on AD-related pathology in mice and provide an overview of the factors that may have contributed to the results reported in these studies. Based on the heterogeneity of these animal model studies and given that the human population itself is quite disparate, it is likely that people will benefit most from individualized nutritional plans based on their medical history and clinical profiles.

## Introduction

Over the past century, life expectancy in the United States has increased dramatically due to successful treatments for serious illnesses such as cardiovascular disease and cancer [1]. However, a longer life expectancy leads to a larger elderly population, which is at high risk of dementia [2]. Therefore, prophylactic and therapeutic approaches for dementia are in high demand. Alzheimer’s disease (AD) is the most prevalent form of dementia, which affected more than 6 million people in the United States in 2021 and is a top 10 cause of death [3, 4]. The number of patients is projected to rise to 13 million Americans by 2050. Despite all the time and resources invested in fighting AD, it still cannot be prevented, cured, or even slowed [4]. Currently there are 6 FDA-approved pharmacological therapies for AD, but they are not curative and only offer a modest clinical benefit [5].

AD is a complex multifactorial disease [6], and its symptoms vary greatly among patients, but commonly include difficulty remembering recent events and names, impaired decision making, and behavioral changes [3]. Histopathological hallmarks of AD include extracellular deposits of beta-amyloid (Aβ) in the form of senile plaques and intracellular inclusions of hyperphosphorylated tau protein in the form of neurofibrillary tangles [3]. AD is also characterized by neuroinflammation, profound neuronal loss, and vascular abnormalities [3].

To identify novel therapeutic approaches for AD, we need a better understanding of genetic and environmental risk factors that contribute to the development of the disease. Although most cases of AD are sporadic, 1% of cases can be attributed to familial mutations in either amyloid precursor protein *(APP)* or presenilin 1 or 2 *(PS1, PS2)*. Familial AD typically manifests itself in patients under 65 years-of-age [7]. Besides age, the largest risk factor for sporadic AD is carrying the E4 allele of the apolipoprotein (*APOE)* gene [8]. Several lifestyle factors also have been linked to the development of sporadic AD. For example, people with more years of formal education and/or a mentally stimulating job or those who stay socially engaged are less likely to develop AD [9, 10], although it is unclear how these factors contribute to reduced risk. Furthermore, history of a traumatic brain injury or poor cardiovascular health, such as hypertension or obesity particularly in mid-life (defined as 40–59 years in most studies), has been shown to increase AD risk [11–15].

**Fig. 1:**
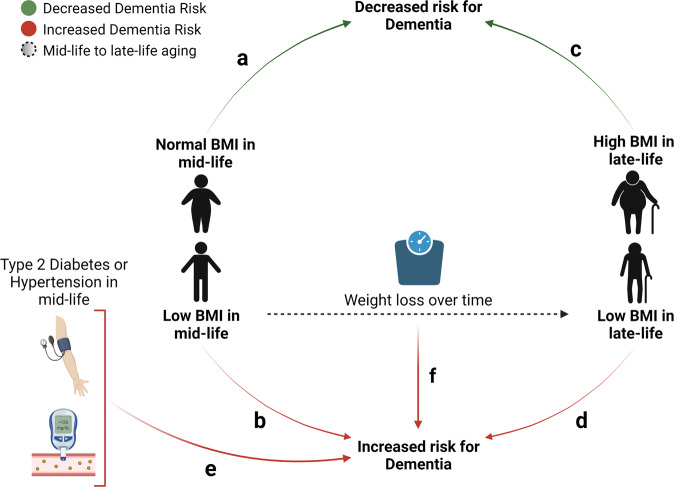
Schematic of obesity paradox, revealed in retrospective clinical studies. A retrospective study conducted on 2 million people over 40 years-of-age showed that underweight people had the highest risk for dementia compared to individuals with a healthy weight in mid-life (**a** vs. **b**), while overweight individuals had the lowest risk for developing AD in late life (**c** vs. **d**) [17, 18]. This observation conflicts with the evidence that cardiovascular risk factors, including hypertension and type 2 diabetes, both commonly linked to obesity, strongly associate with an increased risk for developing dementia (**e**) [11–15]. This paradox is even more complex with the reported finding that more than a 10% loss in weight within 5 years between mid- and late-life is also associated with a 50% increased risk of developing dementia (**f**) [18].

A meta-analysis concluded that by 2050, AD prevalence will be 9% higher in the United States than previously projected due to the drastic increase in the population’s obesity [16]. However, a systematic review of several large well-controlled studies reported that obesity beginning in late life (60 + years) was not associated with higher risk for developing AD [17]. This study also concluded that a low body mass index (BMI) in mid-life significantly increased AD risk. With other large clinical studies concluding that in late-life, underweight patients had the highest risk of dementia and overweight individuals had the lowest risk, these mixed findings contribute to the so-called “obesity paradox” (Fig. 1) [18]. Some authors argue that this retrospective association between low BMI in mid-life and late-life dementia can be attributed to reverse causation—a phenomenon that makes higher BMI in mid-life appear protective, when in reality patients with lower BMIs in late-life could be in the process of weight loss, another factor associated with dementia onset [19]. Future studies are needed to investigate why patients with preclinical AD lose body weight and whether avoiding weight loss would prevent conversion to AD or slow disease progression.

**Fig. 2:**
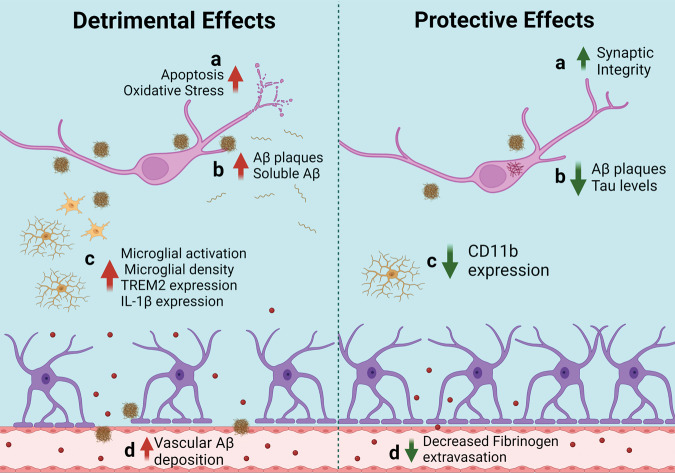
Detrimental and protective effects of HFD consumption observed on AD-related brain pathology in AD mouse models. The effects described throughout this review cover multiple AD mouse models and ages as well as different timelines and types of HFDs. **a** Markers of neuronal dysfunction: Detrimental effects of HFD consumption include increased cortical apoptosis and enhanced whole-brain oxidative stress (not neuron-specific) [34, 35]. Protective effects include increased expression of PSD-95, a marker of axonal and synaptic integrity [61]; **b** Aβ and tau levels: Detrimental effects of a HFD include increased Aβ plaque load and soluble Aβ in the brain [28, 29, 31, 35, 38]. Protective effects include an overall decrease in Aβ plaques [25] and tau inclusions [61]; **c** Microglia: Detrimental effects of HFD consumption include increased microglial activation and density [29], represented by increased expression of TREM2 and IL1β [38, 63]. Conversely, protective effects of HFD administration include decreased microglial activation, characterized by decreased CD11b expression [25]; **d** BBB integrity: In some mouse models, HFD consumption increases CAA (detrimental) [35], while in other models, a HFD protects the BBB as shown by decreased fibrinogen extravasation [24, 25].

The objective of this review is to study the association between dietary fat consumption, obesity, and AD-related pathophysiology in mouse models of AD (Table 1). In animal studies, calorie-dense diets, such as those high in fat, are often used to induce weight gain to determine the association between obesity and AD-related phenotypes. In mouse models of AD, high-fat diets (HFDs) that range between 32–60% fat [20, 21] have been shown to exacerbate AD-related pathology, such as Aβ plaque load and microglial cell activation, and impair cognitive function [22, 23]. However, another study has shown that consumption of a HFD has no effect on Aβ or tau pathology in the brains of other AD transgenic mice, while it is still capable of accelerating cognitive decline [21]. Conversely, other reports show that a HFD improves blood–brain barrier (BBB) integrity and cognition with or without reducing Aβ plaque load in AD mice [24, 25]. Overall, there is no consistent association between diet-induced obesity and AD-related pathophysiology in animal studies which, in part, may be due to technical discrepancies, such as the transgenic AD mouse model used, specific components of the diet, or time course of HFD administration. Here we review how experimental variations could contribute to conflicting reports on how diet-induced obesity affects AD-related phenotypes in mice. The deleterious versus beneficial effects of HFD consumption on AD-related phenotypes in AD mouse models are summarized in Tables 2 and 3, respectively, and compared at a cellular level in Fig. 2.

**Table 1:** Summary of discussed AD models.

AD model	Background strain	Allele(s)	Allele type	Promoter	Aβ/Tau pathology, age of onset	Gliosis, age of onset	Cognitive deficits, age of onset	References
APP/PS1	C57BL/6J	APP (Swedish K670N/M671L); PS1 (L166P)	Transgenic, cDNA	Prnp	6 wks (cortex), 3–4 months (hippocampus)	6 weeks	7 months	Radde et al. [26]
5XFAD	Hybrid C57BL/6 x SJL	APP [Swedish (K670N/M671L), Florida (I716V), and London (V717I)]; PS1 (M146L and L286V)	Transgenic, cDNA	Thy1	2 months	2 months	4–5 months	Oakley et al. [30]
5XFAD	C57BL/6J	APP [Swedish (K670N/M671L), Florida (I716V), and London (V717I)]; PS1 (M146L and L286V)	Transgenic, cDNA	Thy1	2 months	2 months	3–6 months	Jawhar et al. Richard et al. [31, 32}
APP23	C57BL/6	APP (Swedish (K670N/M671L)	Transgenic, cDNA	Thy1	6 months	6 months	3 months	Sturchler-Pierrat et al. [37]
APP ^NL/NL^	C57BL/6	APP (Swedish (K670N/M671L)	Knock-In	Endogenous mouse APP promoter	No Aβ or tau pathology	No gliosis	No cognitive deficits	Saito et al. [39]
APP^NL-F/NL-F^	C57BL/6	APP [Swedish (K670N/M671L) and Iberian (I716F)]	Knock-In	Endogenous mouse APP promoter	6 months	6 months	18 months	Saito et al. [39]
3xTgAD	B6;129 129S4 C57BL/6J	APP [Swedish (K670N/M671L)]; MAPT (P301L); PS1(M146V)	Transgenic, cDNA	Thy1	6 months (Aβ), 12–15 months (tau)	6 months	4 months	Oddo et al. [44]
APOE4	C57BL/6J	Humanized APOE	Knock-In	Endogenous mouse promoter	No Aβ or tau pathology	No gliosis	No cognitive deficits	The Jackson Laboratory, Stock #027894
APP/E4	C57BL/6J	APP (Swedish K670N/M671L); PS1 (L166P); Humanized APOE	Transgenic, cDNA; Knock-In	Prnp Endogenous mouse promoter	6 months	Unknown	Unknown	Nam et al. [52]
Tg2576	Hybrid C57BL/6 x SJL	APP (Swedish K670N/M671L)	Transgenic, cDNA	Prnp	6–10 months	10–16 months	6 months	Hsiao et al. [57]

**Table 2:** Detrimental effects of HFD consumption on AD-related pathologies in AD mouse models.

AD model	Sex	Experimental diet, Cat#, vendor	Control diet, cat #, vendor	Diet consumption start (age)	Diet duration	Aβ and/or Tau pathology	Glia cell activation	Cognitive function	Other	References
APP/PS1	M/F	High fat (42%), TD.88137, Harlan	Standard lab chow (13% kcal from fat), Teklad LM-485, Envigo	4 months	7 months, 17 months	Increased density of Aβ plaques Increased soluble and insoluble Aβ40	Increased microglia density	No effect	Increased social interaction, impaired sensory-motor function	Bracko et al. [29]
	M/F	High fat (60%), Research Diets, D12492	Control diet (10% kcal from fat), Research Diets, D12450J	2 months	7.5 months, 10 months	Increased insoluble Aβ40, increased soluble and insoluble Aβ42	Increased expression of GFAP	Impaired memory	Increased RAGE expression	Walker et al. [28]
5XFAD (C57BL/SJL)	M	High fat (60%), Research Diets	Normal diet (18% kcal from fat), Teklad, Harlan Laboratories	6 weeks	6.5 months	Increased Aβ plaque deposition in the hippocampus	Not evaluated	No effect		Medrano-Jimenez et al. [33]
5XFAD (C57BL/6 J)	M	High fat (60%), D12492, Research Diets	Control diet (10% kcal from fat), D12450B, Research Diets	13 months	10 weeks	Accelerated cerebrovascular Aβ deposition	Not evaluated	Impaired learning and memory	Enhanced brain oxidative stress	Lin et al. [35]
	M	High fat (60%), D12492, Research Diets	Normal chow diet (16.6% kcal from fat), 5K52, LabDiet	3 months	3 months	Not evaluated	Not evaluated	Not evaluated	Increased expression of apoptotic, microglial, and amyloidogenic genes	Reilly et al. [34]
APP23	M/F	High fat (40%), D12079B Western Diet, Research Diets	Normal diet (14% kcal from fat), Prolab Isopro RMH 3000, Lab Diet	11.7 months	3 months	Increased Aβ plaque deposition	Increased TREM2 expression, increase *Trem2* mRNA levels	Impaired spatial learning and memory	Decrease in ABCA1 expression; Increased expression of genes related to immune response, neuronal differentiation, transcription	Nam et al. [38]
APP ^NL/NL^	M	High fat (60%), D12492, Research Diets	Control diet (10% kcal from fat), D12450B, Research Diets	2 months	4 months 16 months	No effect	No effect	No effect	Decrease in hippocampal LTP	Salas et al. [41]
APP ^NL-F/NL-F^	M	High fat (40%), Oriental Yeast	Regular diet (13.8% kcal from fat), CLEA	6 months	12 months	Increased Aβ deposition in the hippocampus	Increased microgliosis in certain hippocampal regions	Impaired cognitive function	Increased oxidative stress in the hippocampus; Decreased expression of transthyretin	Mazzei et al. [43]
3xTgAD	M	High fat (60%), 58G9, Test Diets	Control diet (12% kcal from fat), 58G7, Test Diets	8 weeks	1–2 months, 5–6 months, 13–14 months	No effect	Increased microglia activation	Impaired cognitive function		Knight et al. [21]
	M/F	High fat (60%), 58G9, Test Diets	Control diet (12% kcal from fat), 58G7, Test Diets	2 months	2 months, 6 months, 12 months	Not evaluated	Not evaluated	Increased memory deficits		Martins et al. [64]
	M/F	High fat (60%), D12492, Research Diets	Control diet (10% kcal from fat)	3 months	4 months	Not evaluated	Hypothalamic inflammation in males, increased hypothalamic astrogliosis and IL1β expression in females	Not evaluated	Increased systemic inflammation in males	Robison et al. [63]
	M/F	High fat (60.3%), TD.06414 Envigo Teklad Diets	Control diet (10.5% kcal from fat)	8 weeks	~4 months	Not evaluated	Not evaluated	Spatial memory deficits	Exacerbated brain volume abnormalities	Rollins et al. [46]
	F	High fat (60%)	Normal diet (10% fat)	1 month	4 months	No change in Aβ or tau pathology	Not evaluated	Impaired learning and memory	Increased oxidative stress and neuronal apoptosis	Sah et al. [62]
APOE4	M/F	High fat (45%), D12451, Research Diets	Low-fat diet (10% kcal from fat)	6 months	12 weeks	Not evaluated	Not evaluated	No robust effect on cognitive function	Increased anxiety-like behavior	Jones et al. [50]
	F	High fat & cholesterol (19% butter, 1.25% cholesterol)	Standard chow (3.3% kcal from fat)	12 months	15 months	Not evaluated	Decreased CD68 immunoreactivity in the hippocampus	No effect		Janssen et al. [51]
APP/E4	M/F	High fat (40%), D12079B Western Diet, Research Diets	Normal diet (14.3% kcal from fat)	3.5 months	3 months	Increased Aβ deposition in the cortex and hippocampus	Decreased microglia coverage around Aβ plaques in females	Not evaluated	Sex-specific transcriptome changes	Nam et al. [52]

**Table 3:** Protective effects of HFD on AD-related pathologies in mice.

Model	Sex	Experimental diet(s), Cat #, Vendor	Control diet, Cat #, Vendor	Diet consumption start (Age)	Diet duration	Aβ and/or Tau pathology	Glia cell activation	Cognitive function	Other	References
Tg2576	M	High fat (60%), TD.06414, Envigo Teklad Diets	Control diet (18% kcal from fat), 2018S, Envigo Teklad Diets	2 months	10 months	No changes in Aβ	Not evaluated	Improved spatial learning	Improved BBB integrity; decreased locomotor activity; Increased anxiety-like behavior; decreased brain atrophy	Elhaik Goldman et al. [24]
5XFAD (C57BL/SJL)	M	High fat (60%), D12492, Research Diets	Control diet (10% kcal from fat), D12450J Research Diets	1, 3, and 6 months	5 months	Decreased Aβ plaque deposition	Decreased CD11b expression in the cortex	Improved cognition	Improved BBB integrity	Amelianchik et al. [25]
WT (B6129SF2/J)	M/F	High fat (42%), TD88137, Harlan	Regular chow (13% kcal from fat), 5053, Pico Lab	In-utero (3 weeks)	3 weeks	Reduced pathological tau levels	Not evaluated	Improved learning and memory	Increased synaptic integrity	Di Meco & Pratico [61]

### Deleterious effects of dietary fat consumption on AD pathophysiology in animal models

#### AD mouse models with APP and PS1 mutations

##### APP/PS1 mouse line

The association between HFD consumption and AD has been studied in different AD mouse models, which replicate key pathologies documented in AD patients, including Aβ accumulation, tau hyperphosphorylation, neuroinflammation, and cognitive decline. Many mouse models of AD focus on one aspect of AD pathophysiology—the accumulation of Aβ in the brain. For example, APP/PS1 mice, which express human transgenes for APP with the Swedish mutation (K670N/M671L) and PS1 with the L166P mutation under the control of the neuronal-specific Thy1 promoter, are a widely used model of accelerated Aβ accumulation, which makes them a suitable model to study effects of long-term HFD consumption on AD pathology [26]. APP/PS1 mice begin accumulating Aβ in the cortex as early as at 6 weeks-of-age and in the hippocampus by 3–4 months [26]. By 7 months, APP/PS1 mice begin showing impaired learning and memory [27]. Walker et al. administered a HFD containing 60% fat or a low-fat control diet (LFD) containing 10% fat to 2-month-old APP/PS1 mice and their wild-type (WT) littermates until they were 12 months old [28]. A subgroup of mice was fed a HFD until 9.5 months-of-age and then administered a LFD to determine whether HFD-induced effects could be reversed. In the two-trial Y-maze, HFD impaired memory retention in 12-month-old APP/PS1 mice and this effect was reversed in the mice that switched to LFD. Nest building was also impaired by HFD in 9- and 12-month-old APP/PS1 mice as well as WT mice. HFD increased the levels of insoluble Aβ40 and soluble Aβ42 but not soluble Aβ40 or insoluble Aβ42 [28]. Interestingly, the increased production of soluble Aβ42 was not rescued in mice that were part of the reverse trial, but insoluble Aβ40 and Aβ42 were reduced by the switch from HFD to LFD. Overall, this study demonstrated that long-term HFD consumption leads to cognitive perturbations and alters Aβ accumulation in APP/PS1 mice [28].

Bracko et al. also examined the effect of 42% kcal HFD on APP/PS1 mice starting at 4 months-of-age [29]. These mice showed no memory impairments at 8- or 19 months-of-age but showed impaired sensory-motor function compared to APP/PS1 mice fed a normal chow [29]. HFD administration increased the levels of soluble and insoluble brain Aβ40 in mid- and late-life, while the levels of soluble and insoluble Aβ42 were unaffected in these mice [29]. HFD also increased cortical Aβ plaque density in 21-, but not 11-month-old mice, while hippocampal plaque density was increased by HFD in both age groups. Notably, HFD increased microglia cell density in the hippocampus of both 11-month-old APP/PS1 and WT mice, but this effect was no longer significant at the later age. HFD administration had no effect on the number of occluded capillaries in APP/PS1 mice compared to APP/PS1 mice fed normal chow. Similarly, HFD had no effect on blood flow velocity in either WT or APP/PS1 mice compared to those fed normal diet [29].

In sum, 42–60% HFD consumption affects the levels of Aβ subspecies and increases gliosis in APP/PS1 mice. It may also increase Aβ plaque deposition and induce cognitive deficits, but these changes likely depend on the time-frame of HFD consumption, the composition of the control diet, the behavioral assays employed, and mouse age at the time of testing.

##### 5XFAD mouse line

Recent studies have addressed the effect of HFD consumption on the 5XFAD mouse models (hybrid C57BL/6 x SJL and congenic C57BL6 backgrounds), which overexpress human APP with Swedish (K670N/M671L), Florida (I716V), and London (V717I) mutations as well as mutant human PS1 (M146L, L268V) under the control of the Thy-1 promoter [30]. 5XFAD mice on the hybrid C57BL/6 x SJL background begin accumulating extracellular Aβ at approximately 2 months-of-age and start showing cognitive deficits by 4 months [30]. 5XFAD mice on the congenic C57BL6 background also begin accumulating Aβ at approximately 2 months of age, but the age of onset of cognitive deficits in these mice is more varied at 3- to 6-months-age [31, 32]. Medrano-Jimenez et al. administered a 60% kcal HFD to 6-week-old 5XFAD mice and their C57BL/6 x SJL WT littermates for 8 months [33]. They found that HFD increases Aβ plaque deposition in the hippocampus without affecting learning and memory in the Morris water maze. Reilly et al. administered the same HFD to 5XFAD mice on the congenic C57BL6 background, but they delayed the administration of the diet until the mice were 3 months old [34]. After 2 months, they found HFD increased the levels of circulating lipids and impaired glucose clearance. The authors compared the bacterial composition of feces from 5XFAD and WT littermates and found that while HFD significantly changed microbiome composition in both WT and 5XFAD mice, there was no effect of genotype. An unbiased screen of transcriptomic changes showed that HFD consumption induced the expression of genes associated with the insulin signaling pathway, AD risk, including *Apoe*, and apoptosis. Lin et al. fed 13-month-old 5XFAD mice on the congenic C57BL6 background either a 60% kcal HFD or a LFD for 10 weeks [35]. Interestingly, HFD did not increase body weight in this 5XFAD mouse study, and food intake did not differ between HFD-fed WT and 5XFAD mice, indicating altered metabolism in these aged 5XFAD mice. Similarly, HFD failed to increase blood glucose levels or impair glucose clearance in 5XFAD mice. Despite the lack of physiological changes induced by HFD consumption, HFD-fed 5XFAD mice presented with worse cognitive function compared to 5XAD mice fed the control diet. HFD-fed 5XFAD mice exhibited Aβ deposition around blood vessels of the brain, known as cerebral amyloid angiopathy (CAA), though parenchymal Aβ deposition was unchanged. HFD increased levels of cortical and hippocampal superoxide, NADPH oxidase subunits, and COX-2, suggesting that HFD consumption enhanced oxidative stress in 5XFAD mice. Overall, despite utilizing the same AD mouse model and the same HFD, these various studies did not arrive at the same conclusion regarding the effect of dietary fat consumption on AD-related pathology and cognitive function in 5XFAD mice, likely due to variable timelines of HFD administration.

In summary, in 5XFAD mice on the congenic C57BL6 background, 60% HFD has been reported to increase cerebrovascular Aβ deposition, impair learning and memory, enhance brain oxidative stress, and induce the expression of apoptotic, microglial, and amyloidogenic genes. However, it is difficult to draw parallels between the two studies described above due to the different ages of HFD consumption onset (3 vs. 13 months), varied timelines of HFD consumption (10 weeks vs. 3 months), and differences in the composition of the control diet (ingredient-matched control diet vs. standard chow). Thus, additional studies are needed to elucidate the effect of HFD on AD-related pathology and cognitive function in this widely used AD mouse model.

##### APP23 mouse line

Both 5XFAD and APP/PS1 mouse lines are considered aggressive models of AD since cortical Aβ plaque deposition starts as early as at 6 weeks-of-age [26, 30]. The APP23 line, however, exhibits AD-related pathology around 12 months, which may align with and be more relevant to the human condition. APP23 transgenic mice, which express human APP751 familial Swedish mutation (K670N/M671L) under the control of the mouse Thy-1 promoter, begin accumulating parenchymal Aβ at ~6 months-of-age, although cognitive decline in this AD mouse model precedes extracellular Aβ deposition and can be observed at 3 months-of-age [36, 37]. Nam et al. administered a 40% kcal HFD to 1-year-old APP23 mice for 3 months [38]. HFD consumption impaired learning and memory in the Morris water maze and increased Aβ plaque deposition when compared to APP23 mice fed a normal diet with 16% kcal from fat. Interestingly, while HFD affected male and female APP23 mice similarly, female APP23 mice overall had more Aβ pathology. In line with these findings, HFD consumption increased TREM2 immunoreactivity in the cortex of female HFD-fed APP23 mice, while there was no significant difference between APP23 mice fed either HFD or normal chow, suggesting that Aβ accumulation can modulate TREM2 expression in the brain. RNAseq revealed that HFD led to increased expression of genes related to immune response and inflammation and decreased expression of genes related to neuronal projection and synaptic transmission. Finally, lipidomics analysis revealed that HFD increased the amount of various anionic phospholipids in APP23 mice that play an important role in phagocytosis and apoptosis. Overall, administration of a 40% HFD later in life increases Aβ deposition and impairs cognitive function in APP23 mice, an AD mouse model with abundant Aβ deposition in “middle age”.

#### APP knock-in mouse models

##### APP^NL/NL^ mouse line

Most AD models are transgenic mice that overexpress mutant human genes associated with AD. However, Saito et al. developed an AD mouse model that aimed to overcome potential artifacts introduced by non-physiological overexpression of APP or Aβ [39]. APP^NL/NL^ knock-in mice express APP with a humanized Aβ region and the pathogenic Swedish “NL” mutation (KM670/671NL) under the control of the endogenous APP mouse promoter [39]. As a result, these mice express APP at wild-type levels in the appropriate cell types and brain regions, while also producing pathogenic Aβ40 and Aβ42 [39]. Interestingly, despite elevated production of mutant Aβ, APP^NL/NL^ mice do not show plaque accumulation or cognitive impairment even at 18 months-of-age [40]. Salas et al. administered a 60% kcal HFD or a LFD to 2-month-old APP^NL/NL^ mice for 4 or 16 months [41]. HFD increased body weight, induced hyperglycemia, impaired glucose clearance, and led to peripheral insulin resistance in 6-month-old APP^NL/NL^ mice. Experimental diets did not increase Aβ levels, BACE activity, or tau phosphorylation by 18 months nor was an increase in microglia cell recruitment or astrogliosis in either 6- or 18-month-old old APP^NL/NL^ mice observed. Behaviorally, neither 6- nor 18-month-old APP^NL/NL^ mice fed a HFD presented with impaired contextual or cued fear memory. Salas et al. also used proton magnetic resonance spectroscopy (MRS), a noninvasive neuroimaging technique, to quantify brain metabolites associated with AD in live mice [41]. MRS showed that long-term HFD consumption decreased the ratio of N-acetyl aspartate (a marker of neuronal viability) to myo-inositol (a marker of gliosis). The authors also showed that long-term HFD consumption in APP^NL/NL^ mice impaired long-term potentiation (LTP) but not long-term depression (LTD). In APP^NL/NL^ mice, short- and long-term 60% HFD consumption failed to affect AD-related pathology or cognitive function, although HFD did include a decrease in hippocampal LTP. Therefore, by comparing the results obtained from HFD-fed APP^NL/NL^ mice to those of other transgenic AD mouse lines, it is apparent that the other transgenic AD models may exhibit metabolic aging [42]. Therefore, HFD consumption in these transgenic overexpressing mice might exacerbate already-present metabolic phenotypes, leading to more detrimental effects of AD-related pathology and cognitive function.

##### APP^NL-F/NL-F^ mouse line

In a separate study, it was hypothesized that Aβ deposition prior to the onset of HFD consumption may be a prerequisite for exacerbated development or progression of AD-related pathologies in APP knock-in mouse models of AD [43]. Mazzei et al. used the APP^NL-F/NL-F^ knock-in AD mouse model, which expresses APP with a humanized Aβ region under the control of the endogenous APP mouse promoter. The humanized Aβ sequences in APP^NL-F/NL-F^ knock-in mice contain the Swedish “NL” (KM670/671NL) and Iberian “F” (I716F) mutations. APP^NL-F/NL-F^ knock-in mice start accumulating Aβ as early as at 6 months-of-age and show mild cognitive deficits by 18 months [39]. APP^NL-F/NL-F^ male mice were fed a 40% HFD or a regular diet starting at 6 months-of-age for 12 consecutive weeks [43]. The authors found that HFD increases body weight, fasting glucose levels, and glucose tolerance in both APP^NL-F/NL-F^ mice and their WT littermates. At 18 months-of-age, HFD-fed APP^NL-F/NL-F^ mice showed cognitive deficits by the Morris water maze compared to HFD-fed WT mice and control diet-fed groups. Levels of the postsynaptic protein, PSD95, were decreased and oxidative stress markers were increased in HFD-fed APP^NL-F/NL-F^ mice, when compared to the other three mouse groups. Additionally, 18-month-old APP^NL-F/NL-F^ mice fed a HFD showed increased Aβ plaque deposition in the hippocampus as well as increased insoluble Aβ in the hippocampal extract. HFD also increased glial cell activation in APP^NL-F/NL-F^ mice, but only in the stratum radiatum area of the CA1.

According to the results reported by Salas et al. and Mazzei et al., HFD does not trigger AD-related pathology but instead can exacerbate existing phenotypes. However, a direct comparison between the two studies is not feasible, since diet composition and the timeline of diet administration were varied.

#### AD mouse model with APP, PS1, and MAPT mutations

##### 3xTgAD mouse line

While many mouse models of AD focus on one aspect of AD pathophysiology—the accumulation of Aβ in the brain—there are AD mouse lines that recapitulate several key pathologies of the human condition. For example, the 3xTgAD mouse line contains mutations in APP (Swedish (K670N/M671L)), MAPT (P301L; tau), and PS1 (M146V) [44]. These mice display Aβ and tau pathology starting at 6 and 12 months-of-age, respectively, and cognitive deficits begin at 4 months-of-age [45]. Knight et al. found that 3xTgAD mice that consumed a 60% HFD showed deficits in the Y-maze in later disease stages, but not at earlier stages (3–8 months) [21]. HFD-fed 3xTgAD mice at all ages showed impaired memory in the odor recognition test, a short-term rodent memory test that measures time spent exploring a novel vs. familiar scent. Similarly, HFD impaired memory in the novel object recognition (NOR) test in 3xTgAD mice at all ages and the Morris water maze in 7–8-month-old mice. Interestingly, this effect is transient, as 11–16-month-old HFD-fed 3xTgAD mice no longer showed memory impairment when compared to 3xTgAD mice fed a control diet. Despite its effect on cognitive function, HFD had no significant effect on Aβ accumulation or number of tau-positive cells. The important observation that the authors made was that the effects of HFD on cognition can be either long-lasting or transient [21]. Therefore, discrepancies between different studies examining the association between obesity and AD could be explained not only by the differences in AD mouse models, but also by the choice of behavioral tests and the timeline of diet consumption.

Rollins et al. utilized 3xTgAD mice to investigate the effect of longitudinal HFD consumption on behavior and structural changes in the brain [46]. 8-week-old 3xTgAD mice were placed on either a HFD containing 60% kcal from fat or an ingredient-matched low-fat control diet. At 25 weeks-of-age, HFD did not lead to impaired memory in 3xTgAD mice compared to control-fed 3xTgAD mice in the NOR test, though it did lead to deficits in the Morris water maze. Magnetic resonance imaging (MRI) studies were conducted in 8-, 16-, and 24-week-old mice—at timepoints that correspond to the lack of brain Aβ, the initial accumulation of intracellular Aβ, and the initiation of extracellular Aβ deposition and impaired working memory, respectively. 3xTgAD mice maintained on a HFD show increases in brain volume from 8- to 16-weeks-of-age. However, HFD dramatically decreases brain volume in HFD-fed 3xTgAD mice from 16 to 24 weeks-of-age. Compared to HFD-fed mice, 3xTgAD mice maintained on control diet show only localized increases in brain volume at 8–16 weeks followed by distributed decreases in brain volume at 16–24 weeks, compared to WT mice fed a control diet.

In summary, in the 3xTgAD mouse model of AD, early life (4–8 weeks) consumption of a 60% HFD does not affect Aβ or tau pathology. However, it does increase gliosis and induce cognitive deficits, thus highlighting the importance of long-term monitoring of cognitive health of research rodents, since cognitive changes do not always correlate with Aβ or tau pathology.

#### AD mouse models expressing human APOE

##### APOE4 knock-in mouse line

Most AD mouse models recapitulate human AD pathology as a result of mutations associated with early-onset autosomal dominant AD. However, the majority of AD patients do not harbor these mutations. The biggest genetic risk factor for sporadic AD is APOE4; the E3 allele is associated with normal AD risk [8]. In a study of Caucasian subjects, heterozygous E4 carriers were 3.2 times more likely to develop AD compared to carriers of the more ubiquitous E3 allele [47]. Moreover, homozygous E4 carriers have nearly 15 times increased risk for developing AD [47]. To better study sporadic AD, APOE knock-in mice have been generated where endogenous mouse ApoE is replaced with human APOE isoforms [48]. It should be noted that APOE4 knock-in mice do not develop AD-related phenotypes, such as Aβ deposition or cognitive decline [49]. Jones et al. administered a HFD containing 45% kcal from fat or ingredient-matched LFD to 6-month-old APOE4 mice [50]. HFD increased weight gain, baseline glucose levels, glucose tolerance, and adipose tissue composition in male, but not female APOE4 mice, indicating that HFD causes a sex-dependent metabolic disturbance in APOE4 mice. HFD did not cause any robust changes in cognitive performance [50]. Janssen et al. found that HFD consumption for 3 months had no effect on learning or memory in the Morris water maze in 15-month-old APOE4 female mice. HFD also comparatively decreased CD68 immunoreactivity in the CA1 area of the hippocampus in APOE4 mice, indicating HFD consumption led to decreased neuroinflammation in this line [51].

Overall, chronic consumption of a HFD had no effect on cognitive function in APOE4 mice, although it increased anxiety-like behavior in one study. Given the importance of *Apoe* isoforms for late-onset AD, a tentative conclusion that can be made from these studies is that diet-induced obesity might not confer additional risk for AD in APOE4 carriers. However, since APOE4 mice do not develop any AD-related pathology, these results must be interpreted with caution.

##### APP/E4 mouse line

To evaluate the effect of APOE4 genotype and HFD on Aβ pathology, Nam et al. crossed APOE3 or APOE4 mice to APP/PS1 mice and administered either a normal chow or a sucrose-enriched “Western” diet containing 40% kcal from fat for 3 months starting at 3.5 months-of-age [52]. HFD increased body weight and plasma cholesterol in all mouse groups. However, HFD increased Aβ deposition in the cortex and the hippocampus of APP/E4, but not APP/E3 mice, and this effect was more pronounced in females than males. RNAseq analysis of cortical samples revealed sex-specific transcriptome changes in APP/E4 in response to HFD. HFD increased the expression of genes associated with innate immune response, phagocytosis, regulation of cell migration, and positive regulation of NFkB activity and decreased the expression of genes associated with learning, long-term synaptic potentiation, and protein phosphorylation in HFD-fed female APP/E4 mice. Interestingly, in male APP/E4 mice, HFD increased the expression of genes associated with regulation of transcription and learning. HFD also significantly decreased microglia coverage in female mice only. Specifically, HFD-fed female APP/E4 mice exhibited reduced microglia around large (>200 nm^2^) Aβ plaques. Overall, these results demonstrate that the effects of HFD are influenced by APOE isoform and sex to modulate Aβ deposition, microglia coverage, and cortical transcriptome changes [52]. However, since this study uses a “Western” diet, combining high levels of both fat and sucrose, it is difficult to determine whether dietary fat alone has a similar effect on AD-related pathology in APP/E4 mice. Additionally, further studies are needed to elucidate how HFD affects cognitive performance in this AD mouse model.

#### In-utero studies

The evidence that chronic HFD exposure can affect AD-related pathology and cognitive function is supported by gestational HFD studies. Martin et al. exposed 3xTgAD dams to either a 60% HFD or a LFD during pregnancy and lactation and studied how in-utero exposure to HFD affected the offspring [53]. The offspring remained with their mothers for 21 days and were subsequently weaned and placed on a standard chow diet. Behavioral assessments were performed in 2-, 6-, and 12-month-old female mice, since male mice already showed profound cognitive deficits at 2 months-of-age, regardless of in-utero exposure to diet. Female 3xTgAD mice from HFD-fed dams weighed significantly more at 4 weeks-of-age, when compared to female 3xTgAD from control-fed dams. However, this effect was transient, as there were no differences in the body weight between the two groups at 2 or 12 months. In the Y-maze, there was no difference in the % alternation between 3xTgAD mice from HFD-fed and control-fed dams at 2- or 6 months-of-age, but % alternation was reduced in 3xTgAD mice from HFD-fed dams at 12 months-of-age. Additionally, in-utero HFD exposure affected object recognition memory in 12-month-old 3xTgAD mice. In-utero HFD also increased the number of phosphorylated tau-positive neurons in the hippocampus of 12-month-old 3xTgAD mice while Aβ pathology remained unaffected. Thus, in-utero HFD exposure affected cognitive performance and increased tau pathology in a triple transgenic mouse model of AD, indicating that even short-term exposure to HFD during the critical period of development can affect AD-related phenotypes in 3xTgAD mice.

Several studies also reported the effect of in-utero HFD exposure on cognitive function in WT mice. Tozuka et al. administered either a 60% HFD or standard chow to 5-week-old male and female C57BL/6J mice for 6 weeks until mating, and female mice continued their respective diets through pregnancy and lactation [54]. All mice were fed standard chow starting at lactation day 16 to prevent offspring from eating the dropped HFD before weaning and placed on a normal diet at day 22 on. At 21 days, HFD offspring showed greater accumulation of lipid peroxidation in neuronal cells of the dentate gyrus of the hippocampus. These HFD offspring also had lower mRNA and protein levels of BDNF in the hippocampus when compared to mice from dams fed a normal diet. The changes in BDNF were transient, however, as there were no differences between groups at 70 days. Further studies confirmed that a decrease in hippocampal BDNF expression might be due to oxidative stress. In addition, HFD offspring showed reduced dendritic arborization of new neurons in the hippocampus and had impaired spatial learning in the acquisition phase of the Morris water maze. However, the probe trial did not reveal differences between the groups, indicating intact memory.

To further study the effect of in-utero HFD exposure on synapses in the offspring, male C57BL/6J mice were administered 60% HFD during gestation as described above. Two-photon microscopy was used to study dendritic spines and filopodia in 10-week-old mice [55]. Both formation and elimination rates of filopodia were increased by in-utero HFD exposure, indicating that mice from HFD-fed dams had greater synaptic instability. HFD offspring also showed a persistent increased ratio of filopodia to spines, indicating an impairment in synaptic dynamics and morphology, since the ratio of filopodia to spines is gradually decreased during synaptic development. Offspring from HFD-fed and standard diet-fed dams were then placed on either a HFD or a standard diet after weaning until the age of 8 weeks. Two-photon imaging showed that postnatal HFD exposure induces synaptic instability similar to gestational HFD exposure, and pre- and postnatal HFD exposure does not have an additive effect on the dynamics of dendritic spines and filopodia. However, a significant loss of dendritic spines was only apparent in HFD-fed mice from HFD-fed dams. Interestingly, synaptic instability was also detected in 8-week-old offspring from dams fed a normal diet during pregnancy and a HFD during lactation, indicating that even a short-term exposure to HFD during the critical period of lactation can disrupt synaptic dynamics. These effects were reversed by treatment with ascorbic acid, an antioxidant, in the drinking water, suggesting that this synaptic instability was due to oxidative stress. Yu et al. reported a similar detrimental effect of chronic HFD exposure on learning and memory, which was accompanied by increased serum cholesterol levels, increased brain saturated fatty acid content, and decreased brain polyunsaturated fatty acid concentration [56].

Overall, the results from studies focused on the effects of maternal diet in WT offspring indicate that early exposure to dietary fats may affect cognitive performance in adulthood, which is likely due to brain oxidative stress and, as a result, perturbations in synaptic development. However, future studies will need to determine whether the AD genotype can further exacerbate cognitive function and disrupt synaptic dynamics. Detrimental effects reported to be due to HFD consumption in AD mouse model studies are summarized in Table 2.

### Protective effects of dietary fat consumption on AD pathophysiology in animal models

#### AD mouse models with APP and PS1 mutations

##### Tg2576 AD mouse line

It is important to note that while many studies found a negative or neutral effect of HFD on AD-related pathology and cognitive function, there are several studies that report a protective effect of dietary fat consumption. For example, Elhaik Goldman et al. explored the effect of HFD on Tg2576 male mice, which overexpress human APP with the Swedish mutation (K670N/M671L) under the transcriptional control of the hamster prion gene promoter [24, 57]. Tg2576 mice begin accumulating extracellular Aβ as early as 6 months-of-age but show a rapid increase in Aβ plaque deposition at 10 months [58]. Spatial learning and memory deficits become apparent starting at 6 months-of-age [59]. Tg2576 mice and their WT littermates were administered either a 60% kcal HFD or a control diet from 2 to 12 months-of-age [24]. HFD induced weight gain in both WT and Tg2576 mice. However, Tg2576 mice fed a control diet gained less weight that control diet-fed WT mice. HFD also increased blood glucose levels in both WT and Tg2576 mice at 6 months-of-age but only Tg2576 mice at 11 months-of-age. As expected, HFD increased serum HDL cholesterol levels in both WT and Tg2576 mice. Interestingly, serum cholesterol levels were lower in control-fed Tg2576 mice compared to control-fed WT mice. Compared to other groups, Tg2576 mice fed a control diet showed increased locomotion and time spent in the center of an open field test, indicating hyperactivity and a decrease in anxiety-like behavior. HFD consumption decreased locomotion and increased anxiety-like behavior in both WT and Tg2576 mice, although the effect of HFD on anxiety-like behavior in mice could have been confounded by overall reduced activity in HFD-fed mice [24]. As expected, Tg2576 mice fed a control diet exhibited poor learning in the Morris water maze, but this deficit was partially rescued in the HFD-fed Tg2576 mice. These effects were independent of Aβ pathology, since HFD did not affect the levels of cortical Aβ42. Furthermore, the effect of HFD consumption on BBB integrity was examined by MRI. Although there were no differences between groups at 4 months, there was a trend towards less extravasation in the HFD groups at 8 months. Further analysis showed a significant difference between Tg2576 mice fed control vs. HFD, indicating that HFD increased the integrity of the BBB in Tg2576 mice. At 12 months-of-age, Tg2576 mice showed greater extravasation when compared to WT mice, and there was a trend for greater extravasation in mice fed a control diet when compared to HFD-fed mice. Finally, HFD significantly reduced ventricular volume in Tg2576 mice when compared to Tg2576 mice fed a control diet, indicating a protection against brain atrophy with HFD feeding. This difference was, however, no longer significant at 12 months. Taken together, these results suggest, contrary to some previous reports, that dietary fats might have a protective effect on AD-related pathology and cognitive function. A previous study evaluated the metabolic health of Tg2576 mice and concluded that this AD mouse line shows decreased weight and adiposity, low-plasma leptin levels, and increased energy expenditure at 3 months-of-age, before Aβ pathology begins [60]. These metabolic alterations are accompanied by disturbances in hypothalamic leptin signaling. Metabolic disturbances and hypothalamic changes progress as Aβ burden increases. These results indicate that Tg2576 mice have a pathological metabolic phenotype that can potentially be corrected by long-term HFD consumption, protecting mice from cognitive decline as well as BBB disruption, as discussed in Elhaik Goldman et al.

##### 5XFAD mouse line

Amelianchik et al. reported similar neuroprotective findings in 5XFAD mice [25]. Consumption of 60% kcal HFD for 20 weeks starting at or before 3 months-of-age reduces Aβ plaques in the retrosplenial cortex (RSC) and hippocampus of male 6- and 9-month-old 5XFAD mice on the hybrid C57BL/6 x SJL background. HFD consumption also significantly reduces Aβ throughout the whole brain of 6-month-old 5XFAD mice. In 6-month-old mice, HFD reduces CD11b-positive microglia in the cortex. Additionally, HFD improves memory in fear conditioning and NOR behavioral paradigms in 6-month-old 5XFAD mice. Cognitive performance in the NOR test was similarly improved by HFD in 9-month-old 5XFAD mice that did not start HFD consumption until they were 3 months-of-age. However, delaying the onset of HFD administration until 6 months exacerbated Aβ deposition throughout the whole brain, while Aβ deposition specifically in the RSC and the hippocampus of male 5XFAD mice was not significantly affected by HFD. HFD consumption did little to rescue cognitive deficits in 5XFAD mice that did not start consuming HFD until they were 6 months old, since HFD did not affect object recognition memory in the NOR test in 11-month-old mice. Importantly, HFD improved BBB integrity in 5XFAD mice regardless of the age of onset of HFD consumption, since HFD significantly reduced fibrinogen extravasation from blood vessels into the brain parenchyma in 6-, 9-, and 11-month-old mice, independent of Aβ pathology. Thus, similar to what was reported by Elhaik Goldman et al. [24], chronic HFD consumption exerted a protective effect on AD-related phenotypes, reducing BBB permeability and improving cognitive performance in 5XFAD mice, despite the differences between the two transgenic mouse models. However, Amelianchik et al. also reported a decrease in Aβ pathology after chronic HFD consumption starting at earlier ages, prior to the onset of abundant Aβ pathology, indicating that the timeline of HFD consumption plays a critical role in how HFD affects AD-related pathology [25].

#### In-utero studies

Recent evidence also shows that in-utero exposure to dietary fats might improve cognitive aging and markers of age-related pathology. Di Meco & Pratico (2019) fed B6129SF2/J WT female mice either a 42% kcal HFD or standard chow during gestation [61]. Both the lactating dams and offspring only received regular chow post-partum. The authors then investigated the effect of gestational HFD exposure on cognitive function and brain health in 18-month-old WT offspring [61]. 18-month-old WT mice that received HFD during gestation had increased body weight compared to mice exposed to regular chow in-utero, however there were no differences in blood glucose levels or glucose tolerance. Although gestational HFD did not affect cognitive performance in the Y-maze, HFD-exposed mice showed increased freezing behavior in the cued recall phase of the fear conditioning test, indicating improved amygdala-dependent fear memory. In the Morris water maze, gestational HFD exposure improved learning and memory. This improvement in cognitive performance was accompanied by increased levels of PSD95 in the brains of HFD-exposed mice, indicating better synaptic integrity. WT mice exposed to HFD in-utero also showed a reduction in total tau as well as aggregation-prone and pathogenic tau without changes in tau phosphorylation [61]. Finally, gestational HFD exposure decreased the activation of caspase-3, an enzyme that cleaves tau and primes it for aggregation. Thus, contrary to what has been reported by others, HFD exposure during gestation but not during lactation may exert a beneficial effect on brain health as well as on AD-related pathology and cognitive function.

In sum, some studies indicate that consumption of a HFD may be beneficial to brain health and protective against AD-related pathologies (Fig. 2 and Table 3). Additional studies are still needed to determine the most advantageous HFD component(s) and consumption timeframe for providing therapeutic benefit rather than an adverse outcome.

### Limitations

As evidenced from the studies described above, some reports focused on either male or female mice when investigating the effect of HFD on AD-related pathology and cognitive function, as prior evidence indicates significant baseline Aβ accumulation differences between sexes in certain AD mouse models [21, 62]. Awareness of sex as a biological factor can produce more consistent data on sex-dependent pathology, but without using both sexes and analyzing them separately, comparisons of different investigators’ results can be difficult. Sex differences in HFD-fed AD mouse models have been classified through various experiments investigating insulin resistance, systemic metabolism, Aβ accumulation, inflammation, and cognitive impairments. Robison et al. found that HFD-fed 3xTgAD female mice gained a higher percentage of weight and exhibited more subcutaneous and visceral fat than HFD-fed WT females or HFD-fed 3xTgAD male mice. Female mice also had more severely impaired glucose tolerance and a higher baseline leptin level than WT females and HFD-fed 3xTgAD males [63]. Conversely, males exhibited increased systemic inflammation and increased microglial activation in the hypothalamus, suggesting sex-dependent differences in HFD-induced inflammatory responses. Cognitive decline is suggested to be worse over time in females, as control diet-fed female 3xTgAD mice performed worse in Y-maze experiments compared to their male counterparts at 14 months-of-age [64]. Taken together, these studies highlight that HFD might affect male and female mice differently. Using mice of only one sex or pooling data from male and female mice for the sake of increasing statistical power can lead to results that are inconclusive, misleading, and difficult to interpret.

Another potential limitation of the studies summarized in this review is comparing results obtained from HFD-fed mice to results obtained from mice fed standard grain-based diets, usually referred to as “normal chow”, “regular chow”, or “standard diet”, as opposed to an ingredient-matched control diet. Standard chow diets often contain ground corn, ground wheat, ground oats, fish meal, and other unrefined ingredients [65]. In addition to micronutrients provided inherently from these ingredients, grain-based diets also contain premixes of vitamins and minerals. However, the formula and the levels of nutrients of grain-based diets may be kept proprietary by vendors and change over time. Grain-based diets often contain non-nutrients, including pesticides, heavy metals, genetically modified grains, polychlorinated biphenyls, polychlorinated dibenzo-p-dioxins, and dibenzofurans [66]. In contrast, purified ingredient control diets use refined ingredients (casein, corn starch, sucrose, cellulose, soybean oil, etc.) and have minimal batch-to-batch variability, few non-nutrient chemicals, and have been shown to better match control diets in terms of macronutrients and minerals. When diets are not well-matched, phenotypic differences may be due to any number of dietary differences, not only the fat content of the diet. This is a pervasive issue that stems from the low cost of standard grain-based mouse chow. In fact, 41% of studies surveyed in 2016 presented HFD mouse studies using an inappropriately matched “normal chow” [65].

Another significant limitation is the comparison between transgenic mice and WT mice of the same strain that are not genetically identical or that have not been subject to the same environmental conditions. For example, Lin et al. used 5XFAD mice on the congenic C57BL/6 J background but rather than using WT littermates as controls for the study, WT C57BL/6J mice were purchased [35]. C57BL/6 strains differ significantly, and the differences between various B6 substrains are often larger than the differences between C57Bl/6 mice and other inbred strains such as B10 [67]. Additionally, using strains from other colonies means that the mice also differ epigenetically and are subject to environmental factors that cannot be effectively controlled. In at least two of the studies summarized in this review, the 5XFAD mouse model on the hybrid C57BL/6 x SJL background was used to study how HFD affects AD-related pathology and cognitive function [25, 33]. As this model is maintained by backcrossing transgenic animals to a B6SJLF1 hybrid at every generation, C57BL/6J and SJL/J content is segregating in the progeny of these animals. According to Jackson Laboratory, mice produced from this cross could be genotypically heterozygous, homozygous, or WT for various mutations, including the Trem2^S148E^ allele. To minimize concerns related to allele segregation and the high variability of the original hybrid background, the 5XFAD line was also made on the congenic C57BL/6 J background. However, both lines are still in use. At the moment, the best solutions to bypass these problems are to be conscious of the influence that genetic background might have on the observed phenotypes and to use WT littermates, ensuring equal genetic and environmental influences.

Finally, a major limitation of these studies is the variability between AD mouse lines used. Various transgenic mouse lines express mutated human *APP* and/or *PS1* and/or *MAPT* under the control of various promoters, introducing a significant degree of variability into the pathophysiology, expression levels and expression patterns of pathogenic proteins, and the progression of cognitive decline. In addition, some models are poorly characterized in terms of their sensitivity to behavioral testing, the amount of AD-related pathology, and the extent of synaptic damage, making absolute comparisons between models difficult. The development of knock-in AD mouse lines solved some of the issues associated with transgenic AD mouse lines, as they express mutated human *APP* at physiological levels and under the control of the endogenous mouse promoter. However, it is important to point out that AD-related pathology and cognitive decline in knock-in AD lines develop only after the knock-in of a combination of multiple mutations, which occur in a very small subset of patients with a familial disease form and which typically do no co-occur in individuals. Thus, with AD being almost a uniquely human disease, it is important to further validate the most relevant AD mouse models in order to address the impact of HFD on AD pathophysiology in a way that would be meaningful for clinical practice.

### Future studies

Given the limitations of transgenic AD mouse models described above and by others [68], more studies in knock-in AD mouse lines are needed in order to investigate the effect of HFD on AD-related pathology and cognitive function in a more systematic fashion, including the appropriate use of control animals and ingredient-matched control diets, relevant behavioral tests administered at appropriate ages, and quantitative methods of measuring AD-related pathology and synaptic damage. Furthermore, future studies will need to focus on biological mechanisms underlying either detrimental or advantageous effects of HFD in AD mouse models. For example, Walker et al. hypothesized that increased levels of Aβ could be explained by failure to clear Aβ or disrupted Aβ transport between cerebrospinal fluid (CSF) and the brain (Fig. 2) [28]. Since one of the proteins that mediates Aβ transport into the brain is receptor for advanced glycation end products (RAGE), a transmembrane protein, which is part of the immunoglobulin superfamily [69, 70], Walker et al. examined the levels of RAGE in the hippocampus of HFD-fed APP/PS1 mice. They found a significant upregulation in RAGE by both HFD and the AD genotype, with the most prominent result in HFD-fed APP/PS1 mice, thereby indicating that increased brain Aβ levels might be due to increased RAGE expression. However, a previous study reported RAGE upregulation in blood vessels of 6-month-old APP/PS1 mice fed a 32% HFD for 8 weeks, yet there was no change in Aβ plaque load [23].

Another potential mechanistic explanation for the detrimental effect of HFD on AD-related phenotypes is increased oxidative stress. Several studies included in this review reported enhanced brain oxidative stress in response to HFD intake in different AD mouse lines (Fig. 2) [35, 43, 62]. Lin et al. attributed cognitive impairment in HFD-fed 5XFAD mice to enhanced oxidative stress in the hippocampus in combination with exacerbated CAA [35]. Sah et al. argued that HFD-induced increase in 4-HNE causes oxidative stress and may facilitate neuronal damage and cognitive dysfunction in 3xTgAD mice [62]. Finally, Mazzei et al. reported that HFD induces the accumulation of 8-oxoG, a marker for oxidative stress, in the granule cell layer (GCL) of the dentate gyrus in APP^NL-F/NL-F^ mice. HFD-fed APP^NL-F/NL-F^ mice also show a reduced GCL volume. Thus, HFD-induced oxidative stress exacerbated by either Aβ accumulation of microgliosis may drive hippocampal atrophy by affecting dendrite stability and architecture [43]. Mazzei et al. also hypothesized that HFD-induced increase in Aβ deposition in APP^NL-F/NL-F^ mice might be due to decreased levels of transthyretin [43], a known Aβ-binding protein that can suppress Aβ aggregation [71]. Other possible mechanisms include transcriptome changes that increase the expression of genes related to immune function and inflammation, including *Trem2* [38], IL1β impaired LTP [41], mitochondrial abnormalities [64], and a reduction in microglia barrier in APP/E4 mice (Fig. 2) [52].

Fewer studies report beneficial effects of HFD, but at least two studies described here suggest improvements in BBB integrity, which can protect the brain from further damage induced by blood-derived proteins (Fig. 2) [24, 25]. HFD administration may also enhance synaptic integrity by increasing the levels of PSD95 in adult WT mice exposed to HFD during gestation and lactation (Fig. 2) [61].

Finally, what remains to be studied is the effect of consuming specific dietary fats on AD pathogenesis in mouse models. For example, a recent prospective observational study of aging and dementia among elderly (≥65 years) correlated dietary fat consumption with the risk of developing AD [72]. The intake of docosahexaenoic acid (DHA) and eicosapentaenoic acid (EPA) was associated with a lower risk of AD [72]. The consumption of a “Mediterranean” diet, which is typically high in long chain polyunsaturated fatty acids, also protects against AD dementia [73, 74]. However, it remains unclear whether specific fatty acids contribute to any of the known protective mechanisms, such as an increase in BBB integrity.

### Implications for the human condition

Given the shortcomings of studies addressing the effect of HFD consumption on AD-related pathology and cognitive function, the translational potential of such research remains to be determined. Regardless, the heterogeneity of the findings summarized here is in line with the clinical heterogeneity present in the AD patient population and potentially translatable to the obesity paradox in AD (Fig. 1) that has emerged clinically in recent years. In fact, clinicians often advocate for individualized treatment plans for AD patients, including customized nutritional interventions [75]. This reasoning reflects the complexity of AD pathophysiology, which is often affected by modifiable risk factors. Thus, to achieve AD risk reduction and prevention as well as early intervention, clinical decisions about nutritional interventions should be made based on patients’ individual clinical profiles and include thorough evaluations and nutritional counseling. Such interventions could include nutritional supplements, individualized diet plans, weight monitoring, and/or weight management. HFD studies in AD mouse models show that early dietary interventions, before the onset of abundant Aβ pathology, could be more beneficial. In particular, they might exert a protective effect on the integrity of the BBB. Moreover, as sex plays an important role in how diet affects the metabolic health of males vs. females, the sex of the patient should be taken into consideration when selecting the most appropriate nutritional intervention. Finally, preclinical studies discussed in this review show that long-term and frequent monitoring, including radiographic studies and cognitive testing, are necessary to evaluate the impact of interventions, including those that are dietary in nature, on AD progression.

## Conclusions

Calorically dense diets, specifically those high in fat, provide an opportunity to model diet-induced obesity and investigate the effect of dietary fats on AD-related pathology and cognitive dysfunction in AD mouse lines. However, due to the (1) diversity of AD mouse models, (2) wide range of commercially available high-fat diets, (3) feeding protocols that vary the timelines of HFD consumption, and (4) sex differences in both diet-induced obesity and AD pathophysiology, published studies have failed to yield conclusive results. While many studies report HFD-induced amelioration of AD-related pathology and increased onset and severity of cognitive decline, others report no association between HFD and AD. Moreover, recent studies show that HFD can exert a beneficial effect on AD-related pathologies. The various mechanisms that researchers have observed to be protective or detrimental effects of HFD consumption in mouse models of AD are summarized in Fig. 2. More detailed studies are necessary to uncover the precise involvement of dietary fats in AD pathogenesis and pathophysiology, which may translate into actionable clinical strategies for the human population.
